# All-in-One Gel-Based Electrochromic Devices: Strengths and Recent Developments

**DOI:** 10.3390/ma11030414

**Published:** 2018-03-10

**Authors:** Yolanda Alesanco, Ana Viñuales, Javier Rodriguez, Ramón Tena-Zaera

**Affiliations:** CIDETEC, Paseo Miramón, 196, 20014 Donostia-San Sebastian, Spain; yalesanco@cidetec.es (Y.A.); jrodriguez@cidetec.es (J.R.); rtena@cidetec.es (R.T.-Z.)

**Keywords:** electrochromic, gel electrolyte, semisolid, all-in-one ECD, viologen, conducting polymer, multi-electrochromic, patterned ECDs

## Abstract

Electrochromic devices (ECDs) have aroused great interest because of their potential applicability in displays and smart systems, including windows, rearview mirrors, and helmet visors. In the last decades, different device structures and materials have been proposed to meet the requirements of commercial applications to boost market entry. To this end, employing simple device architectures and achieving a competitive electrolyte are crucial to accomplish easily implementable, high-performance ECDs. The present review outlines devices comprising gel electrolytes as a single electroactive layer (“all-in-one”) ECD architecture, highlighting some advantages and opportunities they offer over other electrochromic systems. In this context, gel electrolytes not only overcome the drawbacks of liquid and solid electrolytes, such as liquid’s low chemical stability and risk of leaking and soil’s slow switching and lack of transparency, but also exhibit further strengths. These include easier processability, suitability for flexible substrates, and improved stabilization of the chemical species involved in redox processes, leading to better cyclability and opening wide possibilities to extend the electrochromic color palette, as discussed herein. Finally, conclusions and outlook are provided.

## 1. Introduction

Electrochromic (EC) materials have attracted growing attention in the last decades because of their proven utility in developing displays [1] (e.g., electronic paper [2]) and smart systems for the automotive and building industries (e.g., rearview mirrors [3,4], helmet visors [5], smart windows [6,7], and climate-adaptive building shells) [8].

Every family of EC materials exhibits strengths that make it more suitable for particular applications. Among them, high stability against UV radiation of the inorganic metal oxides [9,10] (e.g., WO_3_, V_2_O_5_, Nb_2_O_5_, TiO_2_, Ir(OH)_3_, and (Ni(OH)_2_) [11], makes them more proper for use in EC windows designed for energy-saving purposes [8,12,13,14], such as those developed by View Inc. and SAGE Electrochromics Inc. [15,16]. On the other hand, the easier and more cost-effective deposition processes and wider variety of colors provided by organic EC materials such as conducting polymers (e.g., polypyrrole [17], polyaniline [18], and polythiophene and its derivatives [19]) and 1,1′-disubstituted 4,4′-bipyridilium salts, commonly known as viologens, may extend the applicability of EC technology to other applications, such as full-color displays [1,20]. In addition, the fast response times of small organic molecules such as viologens makes them excellent candidates for implementation in antiglare rearview mirrors (e.g., those produced by Gentex [4,21] and Donnelly [22]).

Apart from the EC material, developing competitive electrolytes is crucial to achieving high-performance EC devices (ECDs). The electrolyte ensures ionic transport between the two electrodes to balance the charges that arise from the redox processes. To this end, a model electrolyte of any ECD has to exhibit high ionic conductivity (i.e., between 1 × 10^−3^ and 1 × 10^−4^ s cm^−1^) [23,24], ideal zero electronic conductivity [25], high electrochemical [11,26] and thermal (i.e., up to +60 °C) stability [23], and high transmissivity in the transmissive state [24]. Electrolytes for ECDs conventionally comprise organic polar solvents of high dielectric constant and low viscosity to ease the ion migration [27] in which the electroactive materials can be dissolved [28,29,30]. In spite of the continued use of liquid electrolytes, they exhibit certain weaknesses that may hinder their industrialization [25,31,32,33,34], including the risk of leaking, the presence of bubbles [35], low chemical stability, and safety concerns related to the harmful nature of the organic solvents usually employed. Solid electrolytes first proposed in the literature similarly exhibited some limitations, such as slow switching of the resulting ECDs due to lower mobility of the ionic species into the solid matrix (i.e., solvent-free polymer electrolytes with low ionic conductivities of 1 × 10^−5^ s cm^−1^ [36]) or less transparency, making them unsuitable for ECDs. Aiming to avoid the above-mentioned issues inherent in liquid or solid electrolytes and to find new electrolytes that ensure good interfacing with the electrodes and/or the EC layer [37], a great deal of research has been conducted in the last years in the field of gel and semisolid electrolytes for EC systems, as will be expounded herein.

To benefit the most from EC materials and electrolyte strengths, they must be conveniently incorporated into ECDs along with the rest of the required components. Different device structures with different degrees of complexity have been proposed in the literature [38,39]. However, the most commonly employed device architectures comprise at least two electrode substrates, transparent conducting oxide (TCO)-coated glass or plastic, in a sandwich configuration separated by the electrolyte, along with the electroactive materials. The latter include the EC material and, in most systems, a redox mediator, named the complementary redox pair, which can gain the electrons released by the EC material during the redox process, and vice versa. 

**Figure 1:**
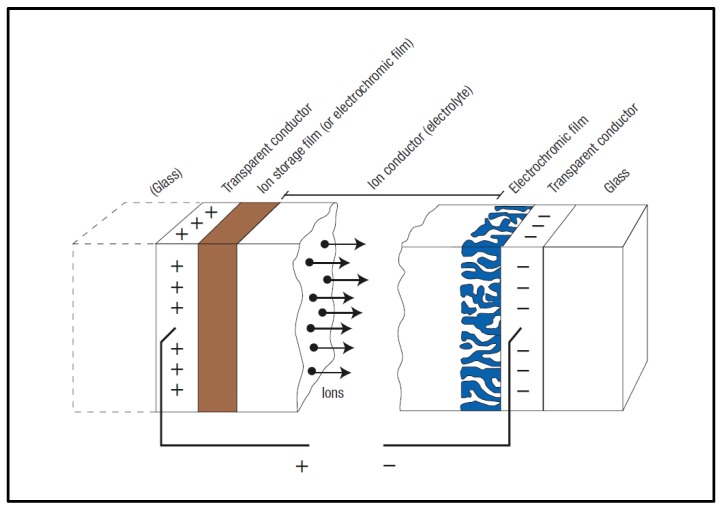
Schematic illustration of a layered electrochromic device (ECD) showing the movement of ions/electrons under an externally applied electric field. Reprinted by permission from [40], copyright (2006) *Nature*.

ECDs can be broadly divided into two main groups: (1) layered-type and (2) all-in-one-type configurations. In this review, layered-type refers to the ECD configuration where the chromophore is deposited on or attached to the working electrode and the electrolyte is incorporated as a discrete layer as follows: glass/TCO/EC layer/electrolyte/TCO/glass. The counter-electrode placed on the opposite side of the device typically comprises an ion storage layer (Figure 1), which can also be EC (i.e., dual-type ECD). Upon applying appropriate external voltage across the two electrode substrates, a redox reaction occurs and electrons and/or ions are interchanged between the EC and the ion storage layer through the electrolyte [40]. A wide variety of layered-type ECDs have been reported based on inorganic metal oxides not soluble in electrolytic media, conducting polymers [38,41,42], or a combination of both [43,44]. Some reported systems comprise inorganic metal oxide films as chromogenic materials (e.g., WO_3_ [45,46,47], V_2_O_5_ [48], TiO_2_ [49], antimony-doped tin oxide (ATO) [50,51], or combinations of them [39]) deposited on one of the electrodes by a high-temperature process (i.e., autoclaving (200 °C) [49], chemical vapor deposition (450 °C) [49], magnetron sputtering (300 °C) [46], thermal evaporation (500 °C) [45], or hydrothermal process (400 °C) [47]. Other fast-switching device architectures [35,52,53] employed these inorganic materials, usually sintered films of TiO_2_ [54,55,56,57] or SnO_2_ [53], just as donor support, where other chromophores (e.g., viologen molecules [2,35,55,56,57,58,59,60,61], among others [53,62]) were chemically absorbed in one or both [53,57] electrodes.

The term “all-in-one” has been used in the literature [63,64] to describe the symmetric device architecture where the EC material and the redox mediator are dissolved in liquid [28,29,30], solid, or semisolid electrolyte [65,66], and the resulting EC mixture (single layer) is sandwiched between two electrode substrates in a very simple configuration (glass/TCO/EC mixture/TCO/glass). This design, although restricted to EC materials that are soluble in the electrolytic media (e.g., viologens), may offer high levels of coloration and ease the fabrication process, making them more interesting from the industrialization point of view. As a result, a variety of work has been and is being published based on this kind of configuration.

The present review outlines all-in-one single-layer ECD architectures comprising gel electrolytes, highlighting some of the advantages and opportunities they offer over other EC systems. First, a brief overview of the types of gel and semisolid electrolytes is included, along with the most recent tendencies reported. Then further strengths of all-in-one ECDs based on gel or semisolid electrolytes are discussed. Some examples are provided of gel electrolytes that not only overcome the drawbacks of liquid and solid electrolytes, but also exhibit easier processability, better suitability for flexible substrates, and improved stabilization of the chemical species involved in the redox processes. That leads to a discussion of enhanced cyclability of the ECDs and opens possibilities for extending the electrochromic color palette. Finally, conclusions and an outlook on all-in-one gel-type ECDs are provided, along with additional investigations desired.

## 2. Gel and Semisolid Electrolytes in ECDs

A great deal of research has been conducted in the last few years in the field of gel and semisolid electrolytes, directly focused on EC systems. Some of the most frequently reported gel electrolytes are based on polymer-salt-solvent systems [23], comprising lithium salts and a polymeric matrix to achieve more solid-like mechanical properties (e.g., PMMA [23,24,26,31,67], PVC [23,33,34], PEO [23,32,38,43] and PAN, [68]), dispersed in a conventional solvent (e.g., propylene carbonate, ethylene carbonate, γ-butyrolactone), exhibiting ionic conductivities up to 4.8 × 10^−3^ s cm^−1^ after the required gelation time (between 3 days [31,32,33,34,43] and 5 days [23]). Among them, PMMA-based electrolytes have gained special importance in ECDs due to their high transparency, good solubility [69], and good ionic conductivity, ascribed to their flexible backbone and amorphous structure, and are still being used [70,71,72].

Other gel-based electrolytes composed of animal protein-derived gelatins in aqueous media with [73] and without [74] the addition of salts, or in organic solvents [75,76], exhibited fast gelation and ionic conductivity values between 1.5 × 10^−5^ and 3 × 10^−3^ s cm^−1^. As a result, these kinds of electrolytes have been suggested as an innovative approach in EC systems based on reversible metal deposition-dissolution [74,77] or inorganic metal oxides [73,75,76].

Recently, cellulose derivatives (e.g., hydroxypropyl cellulose [78], ethyl cellulose [79], and carboxymethyl cellulose [80,81]) have also been used as a natural biodegradable, nontoxic polymeric matrix to obtain gel electrolytes in combination with LiClO_4_ as salt and propylene carbonate as plasticizer [79,80,82]. Accordingly, cellulose-based ECDs comprising inorganic oxides [79,80,81] or conducting polymers [78,82] as a single EC material or in combination with a complementary EC material [78,79,80] have been proposed. Another strategy takes advantage of the abundance and ecofriendly nature of cellulose derivatives and the intrinsic conductivity of laponite [83] to obtain cost-effective, sustainable nanocomposite electrolytes for ECDs [81].

Other approaches, taking advantage of the exceptional properties of ionic liquids (ILs) (e.g., high ionic conductivity, negligible vapor pressure, high boiling point, wide operating potential window, good compatibility with organic solvents, and good chemical and thermal stability [63,84,85,86]), replace the current solvent of the electrolytes in ECDs [63,86]. Accordingly, ILs have been combined with conventional polymers (e.g., polyvinyl alcohol (PVA) [84,87] or PMMA [85,88]), copolymers in well-known ion gels (e.g., PVDF-co-HFP) [89,90,91,92,93,94,95], or gelatin in so-called ion jelly [44], achieving ionic conductivities up to 1 × 10^−3^ s cm^−1^ [85]. Subsequently, an approach based on synthesizing new functional polymers by polymerizing poly(ionic liquid) (PIL) monomers [86,96,97,98,99], in some cases using ILs as liquid solvent [86,97], led to ionic conductivities as high as 1 × 10^−2^ s cm^−1^ [97], and they have been used as promising electrolytes in ECDs.

Lately, a novel kind of ionic fluid known as deep eutectic solvents (DESs) [100], with physico-chemical properties comparable to those of ILs, but with better biodegradability and lower price and toxicity [100], have gained attention. DESs, formed by complexation between appropriate hydrogen bond acceptors and hydrogen bond donors, have been used as ecofriendly solvents or media [101,102,103,104,105] for the synthesis or deposition of EC materials (e.g., conducting polymers [106,107,108] or inorganic metal oxides [109]), and more recently as electrolyte for ECDs [110,111]. The latter, formed by interactions between hydrophilic silica and ethaline [110] or mixing choline chloride with ethylene glycol and glycerol [111], comprised viologens as EC materials and have shown suitable performance.

Apart from the strategies cited above, other innovative approaches have been reported, aimed at obtaining semisolid electrolytes such as those based on organically modified silanes, termed “ormolyte” [48,112,113,114,115,116,117], or obtained by irreversible chemical cross-linked polymers cured by UV radiation [118,119,120,121,122,123] or thermal treatments [63,64,124] before [118,121] or after [119,122,123] being assembled.

All of these approaches, although originally designed to overcome the stated weaknesses of solid and liquid electrolytes, have also contributed to improving the performance of ECDs and offer other possibilities, as discussed below. 

## 3. Processability of All-in-One Gel-Based ECDs

Using gel or semisolid mixtures avoids some assembly difficulties of using liquid or solid electrolytes. Liquid electrolytes are usually introduced by surface capillarity in a previously assembled functional cell, with two small openings placed in opposite corners. Once the liquid electrolyte is put in contact with one of the openings, it ascends through the internal cavity of the cell. However, as the ascent becomes more difficult as filling progresses, due to the growing potential energy of the electrolyte, this method limits the device size and increases the risk of bubbles.

Conversely, assembling solid electrolytes with rigid electrode substrates is very difficult due to their nondeformability. This may limit the electronic contact between the electrode substrates and the EC material [125], leading to colorless areas in the colored state, making it more noticeable in the case of large-area ECDs.

The above-mentioned concerns are avoided when using gel or semisolid electrolytes, as they are easier to manipulate than liquid ones and provide better interaction with the electrode than solid ones (due to their stickiness and/or adhesion [25,26]), thus opening new opportunities for the industrialization stage.

Diverse gel-based all-in-one ECDs assembled through different processes have been reported. Some of the processes used a pre-drying step of the EC composition to remove the co-solvent used during the dissolution or blending process. They include viologen-containing EC mixtures composed of copolymer (e.g., poly(vinylidene fluorideco-hexafluoropropylene) P(VDF-co-HFP)) and an ionic liquid, recently reported by Wang et al. [93] and Moon et al. [94]. In these systems, the gels were obtained by casting or spin-coating their precursor on one of the electrode substrates [93] or another glass surface [94], followed by a drying stage, before covering it with the other electrode [93] or transferring it onto the substrate [94].

**Figure 2:**
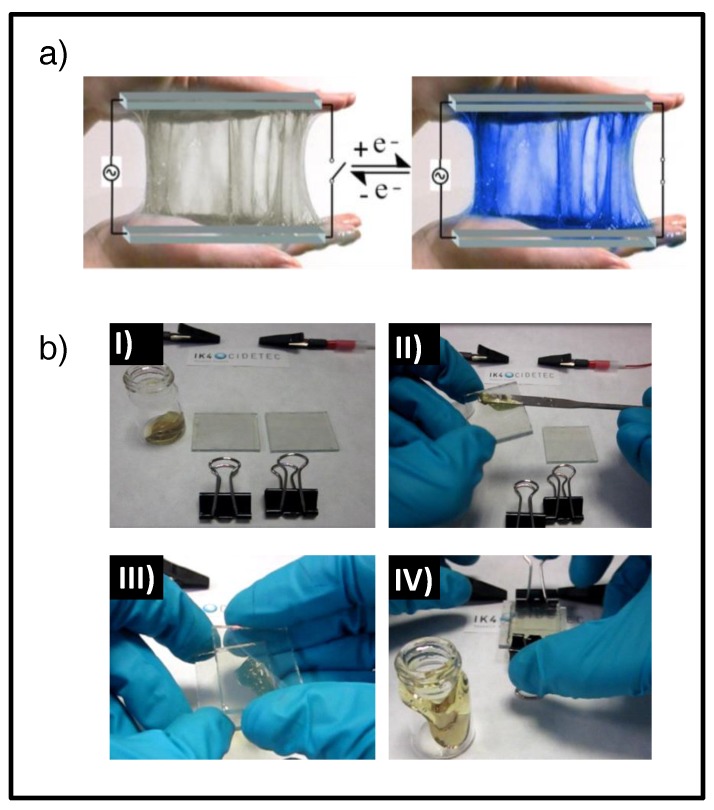
All-in-one gel-based ECDs comprising polyvinyl alcohol (PVA)–borax electrolyte: (**a**) Artistic representation of the device in the bleached (left) and colored (right) state. (**b**) Assembly process: (**I**) Required components: fluorine-doped tin oxide (FTO) glass electrodes, paperclips, and EC gel. (**II**) Application of EC gel to the electrode substrate; (**III**) sandwiching with the second electrode substrate; and (**IV**) clipping the electrodes. Adapted with permission from [126], copyright (2015) John Wiley and Sons, Inc.

Recently, a new gel electrolyte concept based on dynamic covalent chemistries was proven by Alesanco et al. as an ideal polyelectrolyte for easy-to-make ECDs [126]. This kind of polyvinyl alcohol (PVA)-borax gel, obtained by complexation of hydroxyl groups of the PVA chain with borate ions, exhibits rheological behavior about midway between the solid and liquid materials (i.e., non-Newtonian [127,128,129,130], shear thickening [128,129,130,131], and viscoelastic behavior [131,132,133]), as is also observed for different polyhydroxy polymers in the presence of borax [131,134,135]. These attractive rheological properties, with solid-like behavior under high stress but liquid-like character under low stress, allowing it to flow and stretch [128,129] (Figure 2a), can greatly ease the fabrication process. Thus, the assembly process consists of spreading the EC gel on the conducting side of one of the substrates, with a double-sided adhesive tape frame used as a spacer placed along the whole perimeter, covering it with the other electrode substrate (Figure 2b). Then, after applying light pressure, EC gel flows and adapts to the shape of the substrates, providing uniform bubble-free films with excellent contact to both electrodes.

## 4. Durability of All-in-One Gel-Based ECDs

Among the most important requirements for any EC system to be successfully implemented in commercial applications are durability and cyclability (e.g., lifetime over 20 years and around 100,000 cycles for smart windows, and 10,000–100,000 cycles for antiglare rearview mirrors [25]).

The durability of ECDs can be increased by using gel electrolytes, as their elasticity can mitigate the mechanical stress that can arise from manipulation (e.g., bending) and thermal variations, among other factors [25].

**Figure 3:**
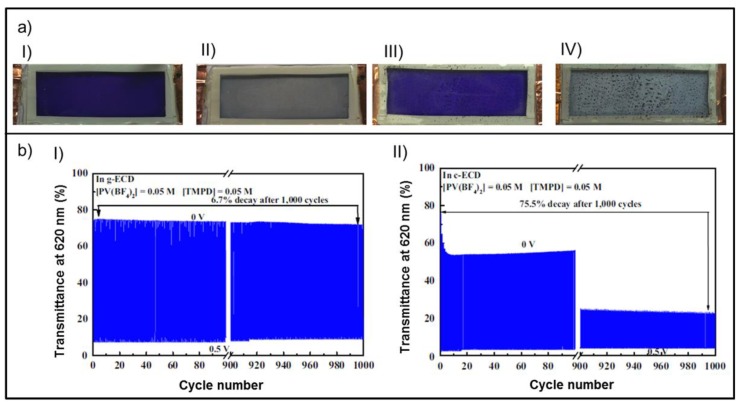
Cycling performance of all-in-one ECDs: (**a**) Polymer-based ECD: (**I**) neutral and (**II**) oxidized states of PProDOT-Ac device (monomer immobilized), with an active area of 1.4 × 4.2 cm^2^ after 10,000 cycles, and (**III**) neutral and (**IV**) oxidized states of control device PProDOT-Me_2_, (monomer not immobilized), showing spotting after 4000 cycles. (**b**) Viologen-based ECD: performance in write-erase ability of the (**I**) g-ECD (gel-type) and (**II**) c-ECD (conventional liquid-type) recorded at 620 nm while the potentials were being switched between 0 and 0.5 V at an interval of 10 s for 1000 cycles. (**a**) Adapted with permission from [123], copyright (2014) American Chemical Society, and (**b**) adapted from [63], copyright (2016), with permission from Elsevier.

With regard to cycling performance, the advantages of using gel electrolytes have been proven for different types of EC materials. In the case of conducting polymers, using gel electrolyte allows immobilization of the monomer to a cross-linkable polymer matrix of the electrolyte, not possible with liquid electrolytes. Following this approach, Otley et al. reported ECDs based on 3,4-propylenedioxythiophene (ProDOT) acrylate derivative as EC monomer and poly(ethylene glycol) diacrylate as cross-linkable polymer, cured photochemically after electrochemical polymerization of the former [123]. When comparing the cycling performance of the as-prepared device (PProDOT-Ac; Figure 3aI and aII) with the one comprising nonimmobilized monomer (PProDOT-Me_2_; Figure 3aIII and aIV), the latter showed a 4% decay of its transmittance change (∆%T) after 500 cycles, accompanied by spotting in the bleached state, whereas the former exhibited improved stability up to 10,000 cycles with a slight decay of 3% of its ∆%T and no visual signs of degradation. These benefits were attributed to immobilization of the unreacted monomeric units, which inhibits their migration onto the electrodes, thus preventing any degradative process.

For viologen-based ECDs, the better cyclability of gel-based all-in-one ECDs compared with those comprising liquid electrolytes has also been proven. Viologens exhibit cathodic electrochromic behavior and three common redox forms: (1) the mainly colorless dication (bipm^2+^), which is the most stable; (2) the radical-cation (bipm^+•^), formed by one electron reduction of the dication; and (3) the neutral state (di-reduced form, bipm^0^), obtained by two-electron reduction of the dication. Despite the fact that highly colored radical-cationic forms are the most exploited in electrochromic devices [136], additional unwanted side reactions or processes have been revealed when using all-in-one device configurations. For instance, it has been proven that the radical-cations of some viologens tend to agglomerate on the surface of the working electrode, forming a deposit [63,119] whose subsequent reorganization may lead to crystallization [63,137,138] and an irreversible bleaching process causing poor cyclability [96]. Conversely, several works agree on the better cycling performance of all-in-one viologen-based ECDs when using gel-type electrolytes. Accordingly, Lu et al. confirmed that poly(ionic liquid) PIL-based gel electrolyte using nonyl viologen as EC material exhibited excellent cyclability, showing a ∆%T of 53.8% after 10,000 cycles, maintaining over 97% of the initial value, whereas it decreased to 45.4% after 4000 cycles in the liquid-type electrolyte [96]. Similarly, other viologen-based gel ECDs with all-in-one configurations have shown better cycling behavior than the corresponding liquid versions, such as the one described by Kao et al. comprising phenyl viologen embedded in a thermally cured cross-linked polymeric matrix [63], or the one comprising two viologens recently reported by Chang et al. [122]. The former showed good cycling performance, with 94.2% of the initial ∆%T remaining after 1000 cycles (Figure 3bI), whereas the Δ%T values of the conventional liquid-type ECD decreased continuously after the first cycle of switching, decreasing by 68% of the initial Δ%T after 1000 cycles (Figure 3bII) [63].

## 5. Suitability of All-in-One Gel-Based ECDs for Flexible Substrates

Another important milestone to expand the potential applicability of EC technology is the development of new systems compatible with most kinds of transparent materials (i.e., plastic or glass) and adaptable to any surface (i.e., flexible).

In this context, all-in-one gel-based ECDs have been proven to meet the flexibility requirement due to two main strengths. First, their assembly process does not involve high-temperature steps, making them compatible with plastic electrode substrates (i.e., tin-doped indium oxide (ITO)/PET). Second, due to the higher viscosity and/or self-standing character of the gel in comparison to liquid EC mixtures, they avoid the risk of leakage and provide more regular distribution (thickness) of the EC mixture throughout the device area, ensuring better homogeneity and avoiding the short circuit between the electrode substrates more likely to occur in flexible, and therefore deformable, systems. 

**Figure 4:**
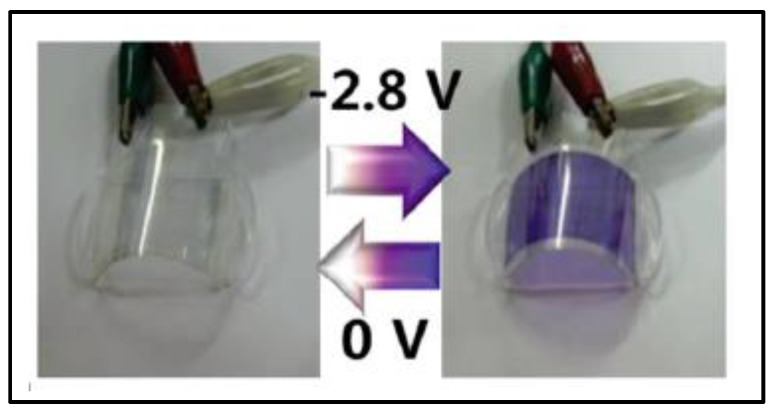
Photographs of reversible performance of a flexible methyl viologen (MV^2+^) graphene quantum dot (GQD) bent ECD with ITO-on-PET in bleached (left) and colored (right) state (−2.8 V). Reprinted with permission from [140], copyright (2014) John Wiley and Sons, Inc.

Accordingly, certain publications have highlighted the manufacture of flexible ECDs comprising all-in-one gel systems. Most of them consisted of an ion gel (IL and a co-polymer) within which the viologens were embedded [94,95,139]. Another innovative approach, reported by Hwang et al., comprised a water solution of PVA together with conductive graphene quantum dots (GQDs) as electrolyte and methyl viologen (MV^2+^) as EC material [140]. They demonstrated that the coloration process that takes places as a result of the donor-acceptor interactions between the π-electron–rich GQD and the π-electron–deficient viologen (MV^2+^–GQD) is homogeneous not only for the flat device, but also for the bent device, providing proper operational stability (Figure 4).

## 6. Low-Energy-Driven All-in-One Gel-Based ECDs

Using low redox potentials prevents some degradation attributed to the use of voltages too close to the electrochemical window of the gel electrolytes [139]. Moreover, low potentials involve lower energy consumption for the switching to occur [85], and therefore to maintain the colored state in electrochromic systems, which require continuous input of electric power to keep it colored (i.e., those that exhibit low memory effect) [94], leading to more competitive ECDs from the industrial and practical point of view [95].

**Figure 5:**
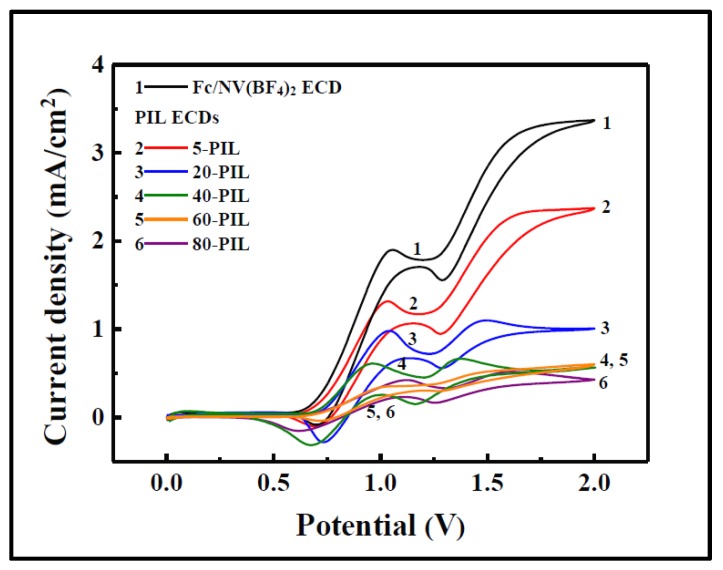
Cyclic voltammograms (CVs) of ECDs based on liquid electrolyte free of poly(ionic liquid) (PIL) (Fc/NV(BF_4_)_2_) and those registered for PIL-based ECDs with different amounts of PIL (from 5 to 80 wt.%) at a scan rate of 50 mV/s. (ITO glass, homemade Ag/Ag^+^, and Pt were employed as the working, reference, and counter electrode, respectively.) Reprinted with permission from [96], copyright (2016) American Chemical Society.

In this context, incorporating IL and other anionic [139,141] and cathodic [141] species into gel electrolytes has been proven to decrease the required redox potentials and the switching times, due to improved ionic conductivity [85,94,95] and lower voltage drop [95]. Accordingly, the work reported by Lu et al. [96] demonstrated that using PIL-based devices not only gives the electrolyte the wanted rubbery character, but also requires lower driven energy due to the additional ions provided by the PIL. Hence, the current densities of the devices decreased as the amount of PIL in the UV-cured electrolyte increased, meaning lower driven energy was required (Figure 5) and making them all lower than that registered for a similar liquid electrolyte free of PIL.

According to several published works based on all-in-one gel ECDs, this is a potential approach to developing low- or even ultra-low-power-consumption EC systems (e.g., e-paper displays [95]) that could also be switched by tiny film batteries, opening new opportunities in the field of printing electronics [94].

## 7. Stabilization of Species in All-in-One Gel-Based ECDs

The improved stabilization of some chemical species not only contributes to better cyclability, as shown in Section 4, but also may open possibilities to extend the electrochromics color palette. In this context, small-molecule EC compounds such as viologens have been widely employed in all-in-one device architectures due to their proven solubility in most common solvents [142]. Several efforts have focused on stabilizing the two reduced forms of viologens (i.e., radical-cation (bipm^+•^) and neutral state (bipm^0^)), along with other combinations (e.g., radical-cation dimer) in a reversible and controlled way. As a result, new all-in-one gel-based EC systems with an enhanced variety of colors have been achieved.

### 7.1. Stabilization of Radical-Cation in Aryl-Substituted Viologens

The radical-cationic form of viologens (bipm^+•^) exhibits different coloration depending on the nature of the substituents on the nitrogen atoms [136]. While the displayed coloration may also depend on the solvent [136], simple alkyl groups provide blue/violet radical cations, while aryl groups generally lead to green coloration.

In this regard, unlike alkyl-substituted viologens, which have been widely reported over the years [65,91,96,142,143,144], aryl-substituted ones have been used less even though they were first synthesized and evaluated more than three decades ago [145,146]. The main reason was the insolubility of their radical-cationic form, detected during the first investigations [147,148], which is more likely to occur in rod-like linear viologens such as aryl-substituted ones when using all-in-one configurations [63].

**Figure 6:**
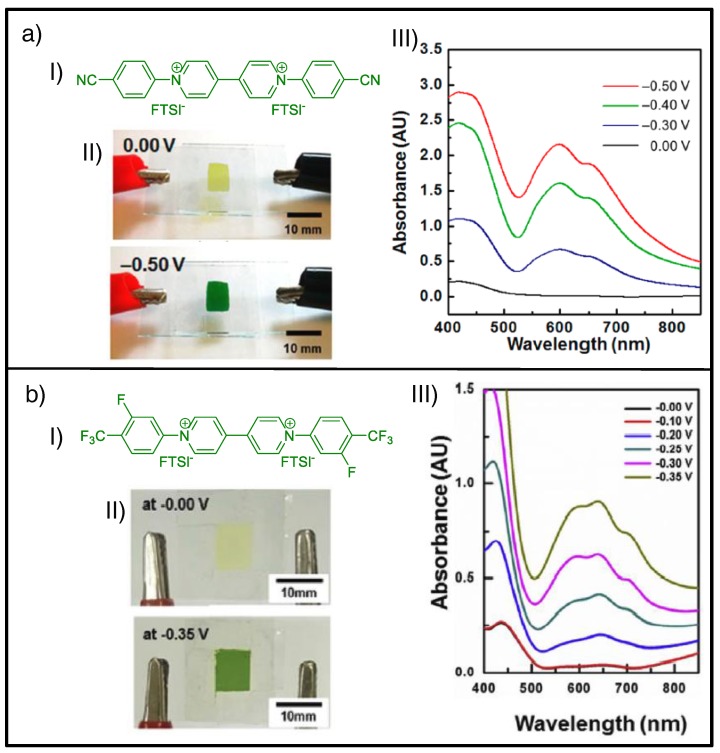
(**a**) ECD comprising 1,1′-bis-(*p-*cyanophenyl)-4,4′-bipyridilium bis(trifluoromethylsulfonyl)imide, (*p*-CV (FTSI)_2_): (**I**) Chemical structure of (*p*-CV (FTSI)_2_); (**II**) photographs of corresponding ECD for bleached state at 0.00 V and green-colored state −0.50 V and (**III**) variation of UV-Vis spectra at different applied voltages. (**b**) ECD comprising 1,1′-bis(3-fluoro-4-(trifluoromethyl)phenyl)-4,4′-bipyridinium bis(trifluoromethylsulfonyl)imide; (**I**) chemical structure of this viologen; (**II**) photographs of corresponding ECD for bleached state at 0.00 V and green-colored state −0.35 V, and (**III**) variation of UV-Vis spectra at different applied voltages. (a) Adapted with permission from [94], copyright (2016) American Chemical Society, and (b) adapted from [92], copyright (2017), with permission from Elsevier.

It took several years before there was renewed interest, and in the last two years a few works have dealt with the use of aryl-substituted viologens in all-in-one configurations aimed at achieving more stable and reversible green-colored ECDs. Although these reports were based on semisolid or gel-like electrolytic matrices, they used different approaches to prevent the aggregation of radical-cationic form. These include the use of dynamic [149] or irreversible [63,64] cross-linked polymer electrolytes or nanofibers obtained by electrospinning dispersed in a liquid electrolyte [99], in which aryl viologens are embedded. Other approaches combined the use of gel electrolytes (i.e., based on ion gels) with the synthesis of new viologens specially designed to avoid precipitation of the radical-cation on the electrode surface, which hinders the bleaching process. Accordingly, Moon et al. replaced the chloride counteranions of the well-known 1,1′-bis-(*p*-cyanophenyl)-4,4′-bipyridilium dichloride (also known as *p*-cyanophenyl viologen dichloride) by the same ones present in IL (i.e., bis(trifluoromethane)sulfonimide (TFSI)) to increase its solubility in the gel electrolyte (Figure 6a) [94]. More recently, Oh et al. additionally modified the molecular structure of the aryl moiety of the latter by replacing the cyanophenyl group with trifluoromethyl and incorporating an additional atom of fluorine (i.e., 1,1′-bis(3-fluoro-4-(trifluoromethyl)phenyl)-4,4′-bipyridinium bis(trifluoromethylsulfonyl)imide), to avoid formation of the quasi-reversible dimer ascribed to π-π stacking of the aryl viologens (Figure 6b) [92]. As a result, green-colored states, characterized by two absorption bands at around 420 and 600 nm in the absorption profiles, were obtained in both cases with no solid aggregations on the electrode surfaces. Growing degrees of green coloration intensity were observed for each system as the applied potential increased, although more green character (i.e., more negative value of a* component of the L*a*b color scale for comparable values of L*) and faster switching times were achieved for the latter [92].

### 7.2. Stabilization of Di-reduced Species

**Figure 7:**
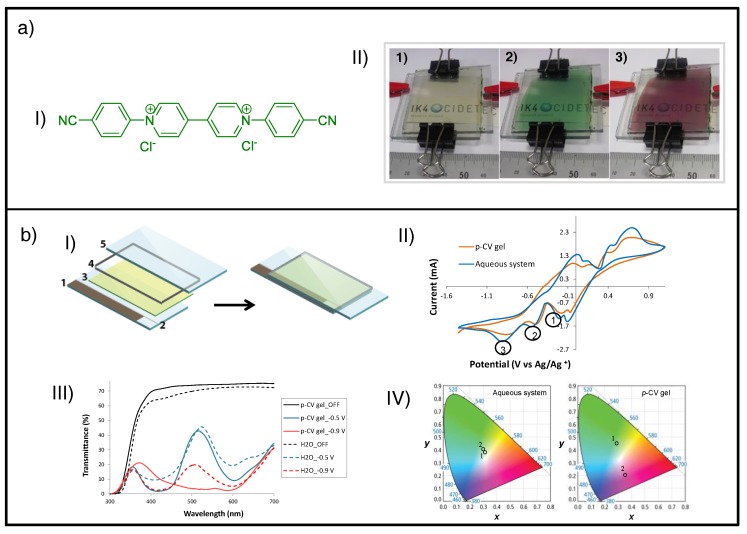
(**a**) Two-electrode ECD: (**I**) Chemical structure of *p-*cyanophenyl viologen dichloride (*p*-CV); (**II**) photographs of an electrochromic device containing *p*-CV viologen in (1) bleached state, and colored states at switching voltages of (**2**) −1.4 V and (**3**) −1.8 V. (**b**) Gel-type (*p*-CV gel) vs. aqueous liquid system (H_2_O) in three-electrode ECD configuration: (**I**) Schematic illustration of the three-electrode ECD configuration including (**1**) Ag/Ag^+^ pseudoreference electrode, (**2**) laser scribing, (**3**) EC gel, (**4**) spacers, and (**5**) TCO/glass; (**II**) cyclic voltammograms at a scan rate of 30 mV/s; (**III**) UV-Vis transmittance response in bleached state and upon applying −0.5 V and −0.9 V voltages; (**IV**) Commission Internationale de I'Eclairage CIE color space plots representing the color coordinates of devices containing aqueous system (left) and *p*-CV gel (right) upon applying (**1**) −0.5 V and (**2**) −0.9 V. Adapted with permission from [149], copyright (2016) American Chemical Society.

The neutral state of viologens (di-reduced form, bipm^0^) has not been exploited in ECDs due to its proven high instability ascribed to its powerful reducing properties [136]. Similar to the aforementioned aggregation and recrystallization problems associated with the radical-cationic form, comproportionation [63,144] can be considered the main issue of the di-reduced species. This redox process between the di-reduced (bipm^0^) and dicationic forms of viologens (bipm^2+^) inhibits the durability of the former, promoting formation of the radical-cation (bipm^+•^) [66]. Although this undesired effect is more likely to happen in all-in-one configurations, the use of gel or semisolid electrolytes was proven by Alesanco et al. to avoid the encounter between the dications and neutral molecules, and therefore comproportionation, due to the higher viscosity of the gel (i.e., 300 Pa s) [149]. Thus, they made stabilization of the di-reduced form of an aryl-substituted viologen (i.e., *p*-cyanophenyl viologen dichloride) possible, achieving a new colored state for more cathodic applied potential (red), in addition to the green coloration shown for lower cathodic voltages (Figure 7a). 

A spectroelectrochemical study using three-electrode device architecture with pseudo-reference electrode (Figure 7bI) suggested a correlation of green and red colorations with the first and second reduction processes of viologen, cathodic peaks 2 and 3 in the cyclic voltammetry (CV), respectively (Figure 7bII). They also compared the electrochemical (Figure 7bII) and electrochromic (Figure 7bIII and bIV) behavior of this gel-type system with the corresponding water-based liquid type and proved that no red coloration was observed for the latter for higher cathodic potentials either in its absorption profiles or in registered color coordinates. Instead, only green coloration was obtained, regardless of the applied potential, as previously observed by other authors for the same viologen in aqueous liquid systems [147,148]. It was ascribed to the radical-cationic form (*p*-CV^+•^) formed by the first reduction of viologen (peak 2 in the CV) and the rapid comproportionation mechanism of the di-reduced form (*p*-CV^0^). Conversely, the gel-type devices allowed stabilization of the di-reduced form, displaying two well-defined colored states (green and red) for different applied potentials.

### 7.3. Stabilization of Radical-Cation Dimer

The radical-cation dimer, formed by a combination of two unpaired electrons of each radical-cation, frequently provides a different colored state from the one displayed by the radical-cation monomer. Even though the radical-cation dimer can be irreversible when all-in-one configurations are used [63,150], some attempts to improve the stability of the dimer in a reversible way have been published (e.g., by host-guest chemistry [151], by covalent preorganization [152], or by using surfactants [153]).

**Figure 8:**
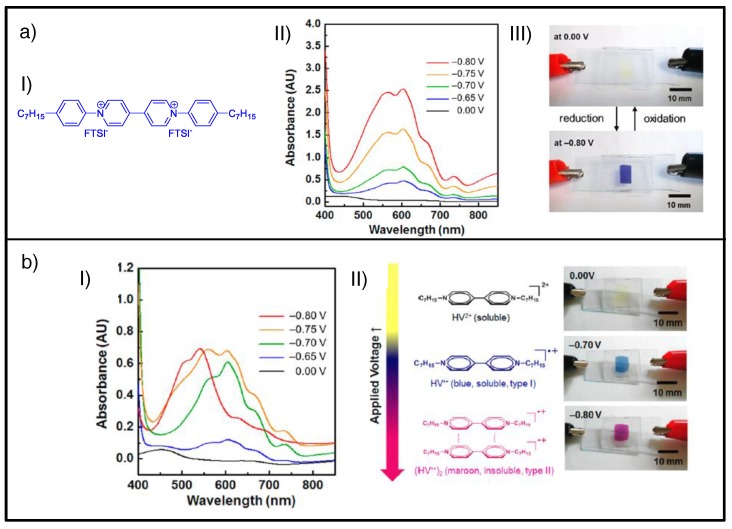
(**a**) EC system based on EC gel comprising BMI-TFSI: (**I**) Chemical structure of heptylviologen bis(trifluoromethane)sulfonamide; (**II**) evolution of UV-Vis spectra at different applied voltages; (**III**) photographs of ECD at bleached (0.00 V) and blue-colored (−0.80 V) state. (**b**) EC system based on EC gel comprising BMI-BF_4_: (**I**) evolution of UV-Vis spectra at different applied voltages; (**II**) photographs of ECD at three different states: bleached (0.00 V), blue-colored (−0.70 V), and red-colored (−0.80 V), along with the corresponding chemical species. Adapted with permission from [94], copyright (2016) American Chemical Society.

Recently, Jordao et al. employed decyl viologen di-iodide as EC material conveniently dissolved into a gel-type composition in an all-in-one configuration and observed two colored states upon reduction, blue and red, associated with the radical-cation monomer and dimer, respectively [154]. Due to the extraordinary stability of the latter, the red coloration disappeared only when high enough oxidation potentials were applied. More recently, Moon et al. achieved stabilization of the radical-cation dimer of the heptylviologen bis(trifluoromethane)sulfonamide (Figure 8aI) by changing the anion of the IL used in the gel composition [94]. They proved that only a blue-colored state (λ_max_ = 605 nm) was achieved when EC gel comprised 1-butyl-3-methylimidazolium bis(trifluoromethylsulfonyl)imide (BMI-TFSI), regardless of the reduction voltage applied (Figure 8aII and aIII). Conversely, when substituting the IL of the gel composition with 1-butyl-3-methylimidazolium tetrafluoroborate (BMI-BF_4_), red coloration was also achieved (λ_max_ = 540 nm) for the same viologen for more cathodic potentials (Figure 8bI and bII), ascribed to the quasi-reversible radical-cation dimer formation. Therefore, simple adaptation of the gel composition leads to a change in the equilibrium between monomer and dimer, allowing two colored states (blue and red).

**Figure 9:**
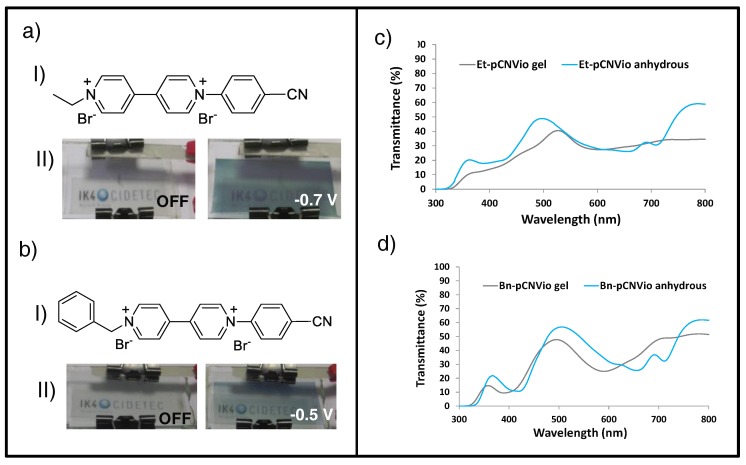
Gel-EC systems based on asymmetric viologens. (**a**) ECD comprising 1-ethyl-1′-(*p*-cyanophenyl)-4,4′-bipyridinium dibromide (Et-pCNVio); (**I**) chemical structure of Et-pCNVio and (**II**) photographs of the corresponding ECD at bleached (off) and gray-colored state (−0.70 V). (**b**) ECD comprising 1-benzyl-1′-(*p*-cyanophenyl)-4,4′-bipyridinium dibromide (Bn-pCNVio); (**I**) chemical structure of Bn-pCNVio and (**II**) photographs of the corresponding ECD at bleached (off) and gray-colored state (−0.50 V). Transmittance profiles of ECDs containing (**c**) Et-pCNVio and (**d**) Bn-pCNVio gels vs. corresponding anhydrous formulations in their colored states. Adapted with permission from [155], copyright (2016) American Chemical Society.

Another clear example of using gel electrolytes to modulate the equilibrium of the radical cation monomer and dimer is from recent work based on 1-alkyl-1′-aryl substituted asymmetric viologens (Figure 9aI and bI) [155]. The latter, with electrochromic and electrochemical behavior about midway between the corresponding symmetric viologens, have been demonstrated to provide neutral tones (i.e., gray, blackish) [156] when conveniently tested in a water-based gel-type electrolyte (Figure 9aII and bII). This report confirmed that using these asymmetric viologens (i.e., 1-ethyl-1′-(*p*-cyanophenyl)-4,4′-bipyridinium dibromide (et-pCNVio, Figure 9aI) and 1-benzyl-1′-(*p*-cyanophenyl)-4,4′-bipyridinium dibromide (Bn-pCNVio, Figure 9bI) in a PVA-borax gel-type electrolyte led to different colored states, and therefore different absorption profiles, from the ones registered for anhydrous liquid systems. Thus, hypsochromic shifts of the maximum contrast wavelength were detected for gel-type formulations along with the new emergent absorption bands at high wavelengths (Figure 9c,d), which translated into grayish-colored states, in contrast to that registered when using anhydrous liquid systems, in which a green hue was dominant (λ_max_ = 650 and 420 nm). Due to its similarity to that reported for better known systems (i.e., 1-1′-alkyl viologens [157,158,159]) this phenomenon was ascribed to the contribution of the radical-cation dimer in gel-type systems, leading to more equal absorption along the most visible wavelength and consequently more grayish-colored states. 

Thus, the use of all-in-one gel-based EC systems also contributes to more colored states, including not only green and red, but also grayish, a very sought-after hue in EC applications [11,160,161,162] due to its better aesthetic adaptability to the surrounding environment and more effective absorption of most of the visible range.

## 8. Versatility of All-in-One Gel-Based Formulations

The capability of EC systems to be easily tailored according to the requirements may extend their implementation in different applications. The two main parameters to assess the electrochromic behavior of EC devices are the level of coloration or transmittance changes and the switching speeds between the colored and bleached states. These two parameters, which can determine the potential use of ECDs, have been proven to be adjustable by changing the amount of electroactive materials, specifically the EC material and the redox mediator.

In contrast to what happens with layered-type devices, where the increment of the level of coloration may be restricted by the limited thickness of the EC layer (due to the presence of cracks or unsuitable voltage drop), all-in-one ECDs have the advantage of being easily adapted by simply modifying the EC mixture they contain. In addition, some gel-type formulations allow more electroactive materials to be used with no signs of precipitation and/or recrystallization, due to their stabilizing properties, explained above. 

Accordingly, as already published for gel-type all-in-one EC systems comprising 1,1′-diethyl-4,4′-bipyridinium dibromide (EtVio) as EC material [126], the EC mixture accepts concentrations of this viologen as high as 25 mmol L^−1^, achieving almost full absorbance in the colored state at the maximum contrast wavelength (550 nm). Alternatively, as the amount of EtVio decreases, the transmittance change equally diminishes, providing EC systems with different levels of coloration, ranging from 69% to 16% for systems comprising 20 and 2.5 mmol L^−1^ of EtVio, respectively.

**Figure 10:**
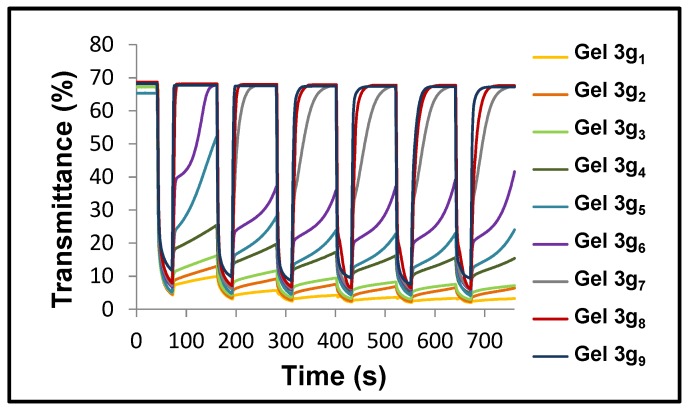
Transmittance changes at the maximum contrast wavelength (550 nm) of ECDs based on gels 3g_1–9_ comprising different amounts of redox pairs (ferro/ferricyanide salts, from 0.4 for 3g_1_ to 6.0 mmol L^−1^ for 3g_9_) while potential steps between bleached (0 V for 90 s) and colored states (−2.3 V for 30 s) were being applied. Reprinted with permission from [126], copyright (2015) John Wiley and Sons, Inc.

Switching speeds were proven to be similarly adaptable by simply modifying the amount of complementary redox pair (potassium ferrocyanide/ferricyanide salts) (Figure 10) [126]. Thus, slow switching times were achieved for compositions comprising low concentrations of this redox mediator, and inversely, very fast commutation times were achieved for those comprising high concentrations of redox pairs (0.4 and 6.0 mmol L^−1^ for 3g_1_ and 3g_9_, respectively).

Thus, these all-in-one gel-based compositions allow the fabrication of not only fast-switching systems useful for applications like antiglare rearview mirrors, but also high-memory-effect systems suitable for smart windows and so on, with modulated levels of coloration.

## 9. All-in-One Gel-Based EC Systems Comprising More Than One EC Material

When using layered-type ECDs, combining more than one EC material in the same device may be complex, as it increases the number of required layers, making the assembly and industrialization processes more difficult. Conversely, the use of all-in-one gel-type ECDs allows easy combination of desired chromophores, which has a direct impact on the color palette.

**Figure 11:**
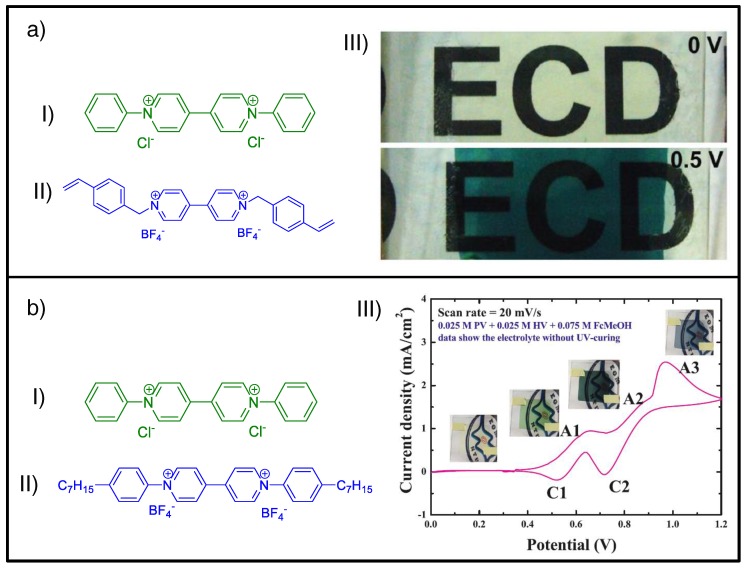
(**a**) ECD based on (**I**) phenyl viologen dichloride (PhVio Cl_2_) and (**II**) vinyl benzyl viologen tetrafluoroborate (BnVio (BF_4_)_2_) and (**III**) photograph of the corresponding ECD at bleached (0 V) and colored (−0.50 V) state. (**b**) ECD based on (**I**) PhVio Cl_2_ and (**I**I) heptyl viologen tetrafluoroborate (HVio (BF_4_)_2_) and (**III**) cyclic voltammogram of the corresponding uncured two-electrode ECD at a scan rate of 20 mV/s. (aIII) Reprinted with permission from [64], copyright (2016) American Chemical Society, and (bIII) reprinted from [122], copyright (2017), with permission from Elsevier.

On the one hand, combining EC materials displaying different colored states in the same device has been demonstrated to provide more neutral colored states than those comprising single-EC material when their contributions occur in the same range of redox potentials. Thus, using bluish- and green-colored EC materials may provide the required grayish-colored state [163], as already reported for complex device architectures (e.g., multilayer [164,165] or multielectrode [166]) based on conducting polymers [166] or small organic molecules (e.g., viologens [163,164] and/or triphenylamine [165]). Following this approach but using all-in-one ECDs, Kao et al. recently reported neutral color panchromatic systems based on the combination of aryl-substituted green-colored viologen (i.e., phenyl viologen dichloride, Figure 11aI) and alkyl-substituted blue-colored viologen (i.e., vinyl benzyl viologen tetrafluoroborate used as EC material and cross-linker, Figure 11aII) in thermally cured gel-type ECDs [64]. In the same way, Chang et al. reported UV-cured semisolid all-in-one ECDs comprising the same aryl-substituted viologen in combination with heptyl viologen tetrafluoroborate (Figure 11bII) as blue-colored EC material, accomplishing more neutral intermediate colored states (Figure 11b) [122].

**Figure 12:**
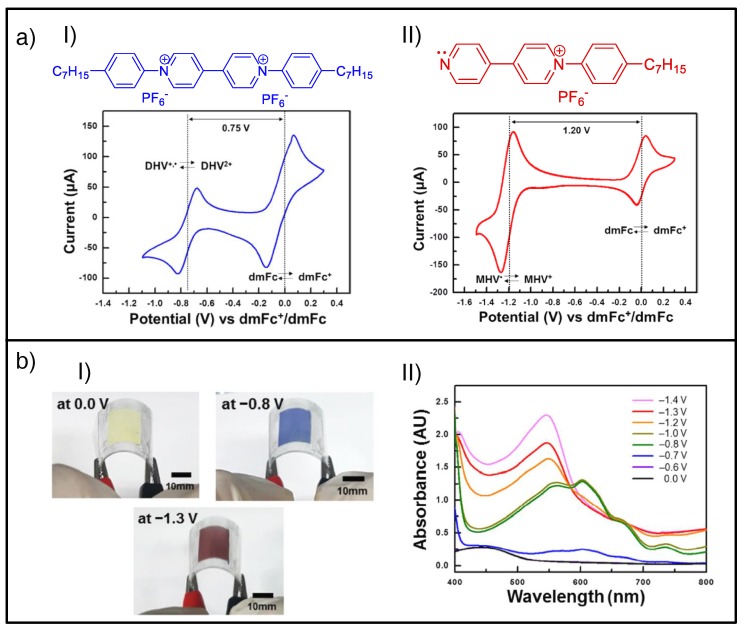
(**a**) Single-viologen–based EC systems: (**I**) chemical structures of diheptyl viologen dihexafluorophosphate (DHV) and (**II**) monoheptyl viologen dihexafluorophosphate (MHV) (top), along with the registered cyclic voltammograms for their corresponding gel-based ECDs at a scan rate of 20 mV/s (bottom). (Pt disk, Ag wire, and ITO-coated glass were employed as the working, reference, and counter electrode, respectively.) (**b**) Blended system comprising a molar ratio of 80/20 (MHV^+^/DHV^2+^): (**I**) photographs of the voltage-tunable flexible ECDs at different applied potentials 0.0 V, −0.8 V and −1.3 V, and (**II**) applied voltage dependence of the absorption spectra. Adapted with permission from [95], copyright (2017) American Chemical Society.

Additionally, when the combined EC materials exhibit different enough redox potentials, two or more distinct colored states may be obtained in the same ECD for different applied voltages. Such is the case with the all-in-one ion gel-based systems described by Oh et al., where the blend of blue-colored diheptyl viologen dihexafluorophosphate (Figure 12aI) with red-colored monoheptyl viologen dihexafluorophosphate (Figure 12aII) led to voltage-tunable bichromic ECDs (Figure 12b) [95].

**Figure 13:**
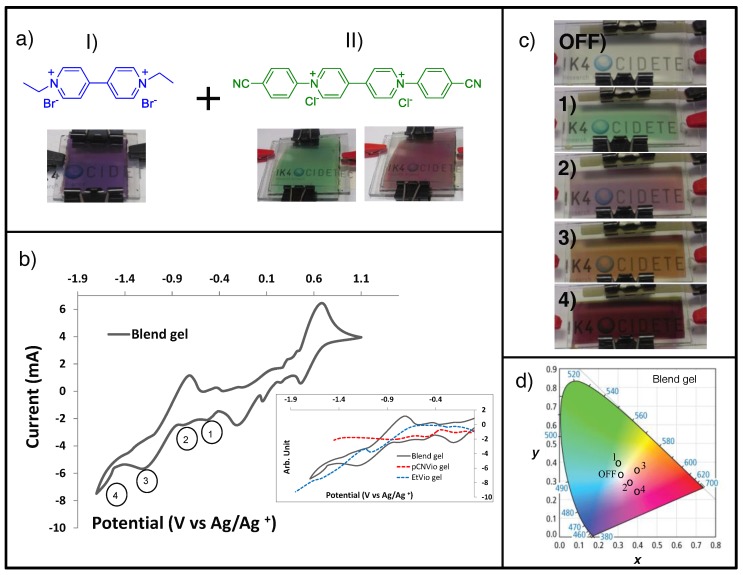
(**a**) Single-viologen–based EC systems: (**I**) chemical structures of employed viologens, monoelectrochromic ethyl viologen dibromide and (**II**) bielectrochromic *p*-cyanophenyl viologen dichloride (*p*-CV) (top), with photographs of the corresponding ECDs at colored states (bottom). (**b**–**d**) Multicolor blend system: (**b**) CV of blended gel and comparison with CVs of *p*-CV gel and EtVio gel separately (inset) using three-electrode ECDs (similar architecture to Figure 7bI at a scan rate of 30 mV/s); (**c**) photographs after applying a switching voltage of 0 V (off, colorless), −0.7 V (1, green), −0.9 V (2, pink-violet), −1.1 V (3, orange), and −1.7 V (4, purple); and (**d**) CIE color space plots representing color coordinates registered at the same potentials. (aI) Adapted with permission from [126], copyright (2015) John Wiley and Sons, Inc., and (aII, b–d) adapted with permission from [149], copyright (2016) American Chemical Society.

Furthermore, if one of the EC materials exhibits more than one colored state, all-in-one multi-ECDs may be obtained by simply blending two EC materials, as already shown [149]. The latter comprised blue-colored ethyl viologen dibromide (Figure 13aI) combined with well-stabilized *p*-cyanophenyl viologen dichloride, which provides green- and red-colored states, as discussed above (Figure 13aII), leading to four well-defined cathodic peaks for the blend (Figure 13b), each providing a different colored state (Figure 13c,d).

## 10. Patterned ECDs Based on All-in-One Gel-Type EC Systems

The mechanical strength and self-standing character of some semisolid or gel-type EC mixtures offer significant advantages in easing the design of patterned ECDs, thus allowing the devices to display information by different techniques.

**Figure 14:**
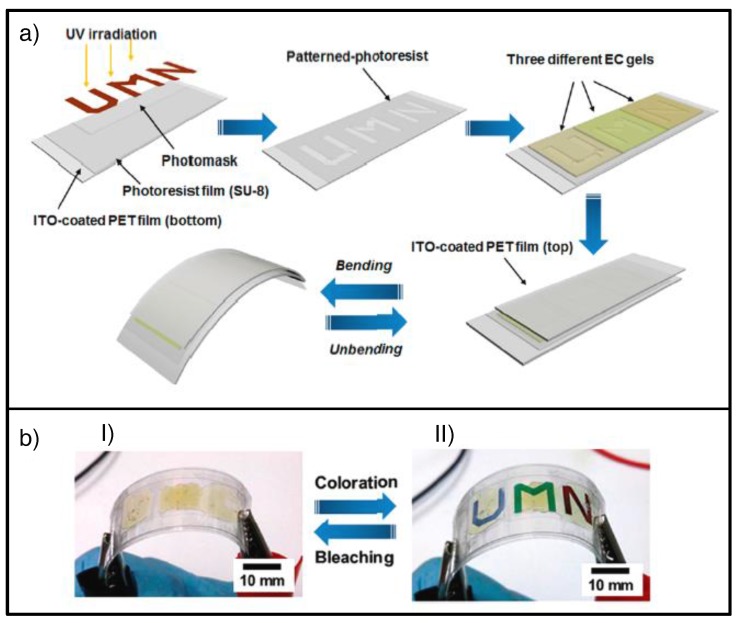
Cut-and-stick method: (**a**) Schematic illustration of fabrication processes for a flexible, patterned, multicolored ECD on plastic sheet; (**b**) photographs of resulting flexible ECDs: (**I**) ECD at bleached and (**II**) colored state, 0.00 and −0.70 V, respectively, with bending. Adapted with permission from [94], copyright (2016) American Chemical Society.

Moon et al. reported all-in-one ECDs based on ion gels using a cut-and-stick method [139]. These EC gels may be cut into particular shapes and then transferred onto electrode substrate, then sandwiched with the other electrode substrate [94]. Besides that, the use of previously photolithographed electrode substrates allows the design of additional well-defined patterns (e.g., letters, numbers, and so on) (Figure 14a). Furthermore, the use of more than one mono-EC gel offering different colored states in the same device led to patterned multicolored ECD, also compatible with plastic flexible substrates (Figure 14b).

**Figure 15:**
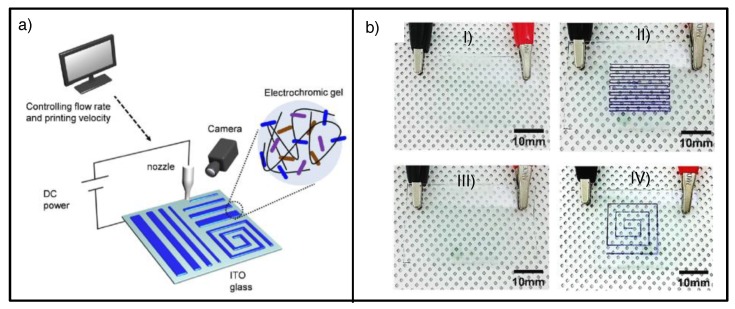
(**a**) Schematic illustration of electrostatic force–assisted dispensing printing and printed EC gel; (**b**) photographs of ECDs comprising EC gels printed by this technique with different shapes: ECDs in their (**I**,**III**) bleached state (left) and (**II**,**IV**) colored state upon applying a redox voltage of −0.8 V (right). Adapted with permission from [90], copyright (2017) American Chemical Society.

More recently, Kim et al. reported an innovative process that provides well-defined directly patterned ECDs without the use of photolithography, also based on ion gels. The rubbery and self-standing consistency of the EC gels allows them to be directly printed on the electrode substrate while maintaining dimensional stability [90]. They used the so-called electrostatic force-assisted dispersing printing method, where controlled applied potential between the electrode substrate and the printer nozzle during the deposition process enhances the dimensional stability and adhesion by electrostatic interactions (between the negatively charged substrate and the positively charged ion gel surface) (Figure 15a). Once the EC gel was printed, it was thermally annealed and subsequently covered with the other electrode, maintaining the well-defined printed shapes in the final ECDs with feature sizes smaller than 1 mm (Figure 15b).

Thus, the use of gel-type EC systems allows the design of patterned ECDs while maintaining the simple device architecture. 

## 11. Conclusions and Outlook

A great deal of research has been conducted in recent years in the field of gel and semisolid electrolytes for EC systems. As pointed out in this overview, they range from the simplest polymer-salt-solvent to IL- or PIL-based compositions, through more ecofriendly solutions such as those comprising gelatin-based cellulose derivatives or deep eutectic solvent systems, at times employing dynamic or irreversible cross-linked networks to achieve more solid-like mechanical properties. Although initially focused on avoiding the issues inherent in liquid electrolytes (e.g., risk of leaking, presence of bubbles, low chemical stability, and some safety concerns) and solid electrolytes (e.g., less transparency or slower switching of the resulting ECDs due to lower ionic conductivity), all of these approaches have contributed to improving the performance of ECDs and possibilities for their use. In addition, the combination of these gel-type electrolytes with all-in-one device configurations has led to more competitive EC systems, with emerging opportunities to boost market entry in commercial applications. These kinds of EC systems have been proven to be easily modulated according to need (e.g., ∆%T and switching times), are suitable for flexible substrates, have enhanced durability, and are low-energy-driven devices, easing the assembly process. All of these features represent a competitive advantage over other EC systems (i.e., liquid-type and solid-type electrolytes or layered-type device architectures), leading to more competitive ECDs, adjustable for diverse applications (e.g., fast-switching or high-memory-effect systems with different levels of coloration), with easy patternability, requiring ultra-low power consumption that could be switched by tiny film batteries and adapted to any surface.

Apart from the expected advantages of gels due to their mechanical properties, this review shows great potential for all-in-one gel-based formulations to enhance the performance of electrochromic devices. Thus, the feasibility of these all-in-one gel-based EC systems to stabilize radical-cation monomers, dimers, and di-reduced species and allow easy mixing of more than one EC material may contribute to extending the chromatic diversity of ECDs, including green, red, and the sought-after grayish coloration, or even multi-EC systems. In this context, it is worth noting a rapidly growing interest in green-colored ECDs, reactivated as a result of the more effective stabilization of aryl-substituted viologens in an all-in-one simple device configuration using gel or semisolid electrolytes.

Despite the mentioned strengths and the fact that gel-type electrolytes may avoid some of the assembly problems when using liquid or solid-type electrolytes on a laboratory scale, the existing upscaling printing or coating processes (i.e., inkjet, dip-coating, slide-bead, gravure, slot-die, or roll-to-roll) have not been studied enough with this kind of gel-type rheology. Once some of these techniques are adapted to gel-type electrolytes, it may open new opportunities for the industrialization stage, meeting the requirements for use in printing electronics.

Additionally, as a future outlook, all redox species proposed for viologens should be further confirmed through more accurate techniques, such as in situ spectroelectrochemistry. The simultaneous use of electron spin resonance, which enables identification of paramagnetic (i.e., radical-cation monomer) and diamagnetic (i.e., di-reduced (neutral) and radical-cation dimer) redox species, and UV-Vis spectroscopy would allow unequivocal correlation between an observed coloration and the specific redox species involved. The confirmation of di-reduced species, specifically aryl-substituted viologens (i.e., *p*-cyanophenyl viologen dichloride), would be of particular interest due to the scarcity of reports dealing with their formation and stabilization in ECDs. This knowledge would meaningfully help in better understanding the redox processes of viologens in general, and aryl-substituted viologens in particular, and is expected to enable development of ECDs with new and improved colored states.
